# Impaction bone grafting for contained acetabular defects in total hip arthroplasty

**DOI:** 10.1186/s13018-023-04154-0

**Published:** 2023-09-11

**Authors:** Mohamed Yousry Abu-Zeid, Mohamed El-Sawy Habib, Sameh Mohamed Marei, Ahmed Nasr-Eldin Elbarbary, Ahmed Ali Ebied, Mohamed Kamal Mesregah

**Affiliations:** Department of Orthopaedic Surgery, Faculty of Medicine, Menoufia University, Shebin-El-Kom, Menoufia Egypt

**Keywords:** Acetabular deficiency, Acetabular defects, Impaction bone grafting, IBG, Autologous bone, Allograft bone, Total hip replacement, Revision hip arthroplasty

## Abstract

**Background:**

Acetabular bone loss is a technical challenge in total hip arthroplasty (THA). This study sought to report the functional and radiological results of acetabular reconstruction using impaction bone grafting (IBG) in patients with acetabular bone deficiency undergoing primary or revision THA.

**Methods:**

In this prospective study, full history taking, preoperative clinical and radiological evaluation, and preoperative planning and templating were performed. The Paprosky classification and the American Academy of Orthopaedic Surgeons classification were used to assess the acetabular deficiencies. Clinical outcomes were assessed utilizing the Harris hip score (HHS) and a 4-question satisfaction questionnaire. Graft incorporation was evaluated in the last follow-up X-rays.

**Results:**

This study included 50 patients with a mean age of 46.7 ± 15.3 years. The THA was primary in 14 (28%) patients and revision in 36 (72%) patients. The mean HHS improved significantly from 28.8 ± 24.1 preoperatively to 76.6 ± 6.1, with a mean follow-up period of 23 months. Overall, 88% of patients were very satisfied. Complete radiological graft incorporation to host bone was achieved in 35 (70%) patients, and the remaining patients had partial incorporation. Complete graft incorporation was associated more frequently with primary THA, autografts, cementless cups, decreased defect size, and decreased graft layer thickness.

**Conclusions:**

IBG for acetabular reconstruction in THA can achieve excellent clinical and radiological outcomes with a low complication rate.

**Level of evidence:**

Level IV.

## Background

The number of total hip arthroplasty (THA) surgeries is growing annually [1]. Orthopedic surgeons must be prepared for the technical challenges of primary and revision surgery, including acetabular bone loss, especially when bone loss compromises acetabular column support [1, 2].

Osteolysis and loosening are still significant complications of THA [3]. Additionally, the loss of bone stock is a critical issue that can negatively affect the outcomes of revision surgery [1].

Identifying the appropriate method of acetabular reconstruction depends on properly classifying acetabular bone loss and evaluating the patient thoroughly [1, 4].

Various strategies can be utilized for acetabular reconstruction in case of acetabular deficiencies, including bulk allografts, bone cement, augments, rings, or cages [3, 5, 6]. Another option is using large cups; however, bone stock can be compromised further by the use of large implants [1, 7].

Impaction bone grafting (IBG) is a reliable biological and mechanical method to restore acetabular bone-stock deficiency [2, 3, 8]. Femoral head autograft or fresh-frozen allograft is morselized into small cancellous pieces, which are then impacted into the defect before inserting the primary or revision acetabular components [9–11]. The impacted graft can restore the bone loss, form a durable scaffold for the acetabular implant, incorporate into the host bone, and undergo gradual remodeling [10, 11].

Cavitary deficiencies can be managed with IBG alone; however, IBG in segmental or combined acetabular deficiencies usually requires supplementary implants such as metal mesh, rings, or cages to close peripheral defects and convert the non-contained into contained defects [10, 12, 13].

Despite good reported clinical results with IBG for acetabular deficiencies, failure of graft incorporation, insufficient cement mantle, and inadequate stability may lead to cup loosening, migration, or subsidence [11, 14].

This study aimed to report the functional and radiological results of acetabular reconstruction using IBG in patients with bone-stock deficiencies undergoing primary or revision THA.

## Methods

This prospective study included patients who had undergone acetabular reconstruction by IBG in primary and revision THA between April 2020 and February 2022. Approval of the Institutional Review Board (IRB) was obtained prior to conducting the study, besides written consent from participating patients.

Inclusion criteria were patients with acetabular defects that needed reconstruction at the time of THA. The primary causes of acetabular cavitary defect were either protrusio acetabuli or acetabulum fractures. Revision causes were cup loosening with acetabular bone defect. The minimum follow-up period was 12 months. Patients with acetabular defects due to primary bone tumors or metastasis were excluded from the study.

### Preoperative assessment

Full history taking and preoperative clinical and radiological evaluation were conducted. Meticulous local examination of the involved hip was done, and scars of the previous operations were documented. Abductor muscle strength was evaluated using the Trendelenburg test and resisted side-lying abduction.

Radiological investigations included anteroposterior (AP) pelvis X-ray, and AP and lateral X-ray views of the affected hip to determine the amount of bone loss of the acetabulum and to classify the defects. Additionally, computed tomography (C.T.) scans were used in patients with complex defects. The Paprosky classification [15] and the American Academy of Orthopaedic Surgeons (AAOS) classification [16] were used to evaluate acetabular bone loss.

Preoperative planning and templating were performed to evaluate the defect size and type of prosthesis, type of bearing surface, method of acetabular reconstruction, and how to deal with LLD. Preoperative leg-length discrepancy (LLD) was evaluated through the difference in distance from the interischial line to the top on the lesser trochanter bilaterally. John and Fisher method [17] for radiological determination of the hip center of rotation was used to determine the presumed center of rotation.

Preoperative venous Doppler was done routinely to exclude DVT in bed-ridden patients. Erythrocyte sedimentation rate (ESR) and C-reactive protein (CRP) levels were obtained.

### Surgical technique

Patients were placed in the lateral decubitus position under spinal or epidural anesthesia. A single dose of 1-g third-generation cephalosporin was administrated intravenously at the induction of anesthesia, followed by 1-g vancomycin intravenous infusion.

The posterior hip approach was utilized, and in revision cases, the incision was usually extended proximally and distally to define tissue planes more easily to release scars and to facilitate extensile exposures when needed.

The femoral head and neck were resected in primary cases, and the existing prosthesis was removed in revision cases. The true floor and the transverse acetabular ligament (TAL) were identified after osteophytes removal. A Steinmann pin was then inserted in the posterior wall and ischium to protect the posterior soft tissues, including the sciatic nerve. Excision of the hypertrophied labrum and remnants of the anterior capsule was then performed.

**Fig. 1:**
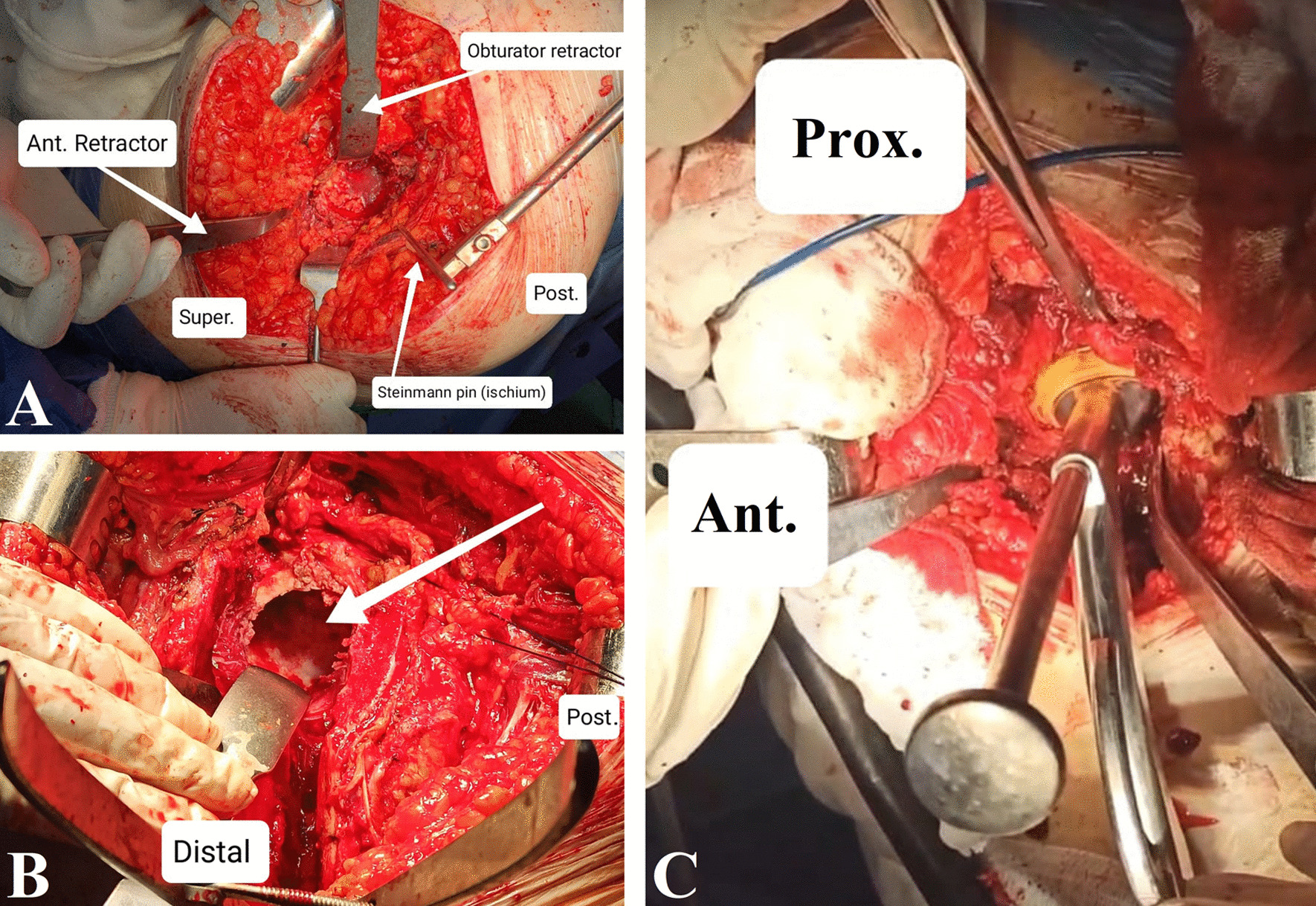
Intraoperative photographs of a primary THA case. **A** Exposure of the acetabulum. **B** Identification of the defect. **C** Acetabular component sizing

Identification of the correct placement of the cup and the site of the acetabular defect before reaming were done. The acetabulum was prepared with gouges and reamers to expose sufficient cancellous bone while maintaining subchondral plate at the periphery of the acetabulum for better mechanical support to the socket. The correct size of the acetabular component or the acetabular reconstruction augment, ring, or augmented dual-mobility cup that achieved maximum bony contact was determined before the sizing of the defect. With the trial cup in place, the defect was assessed, and the proposed position of IBG was determined using the trial components, Fig. 1.

**Fig. 2:**
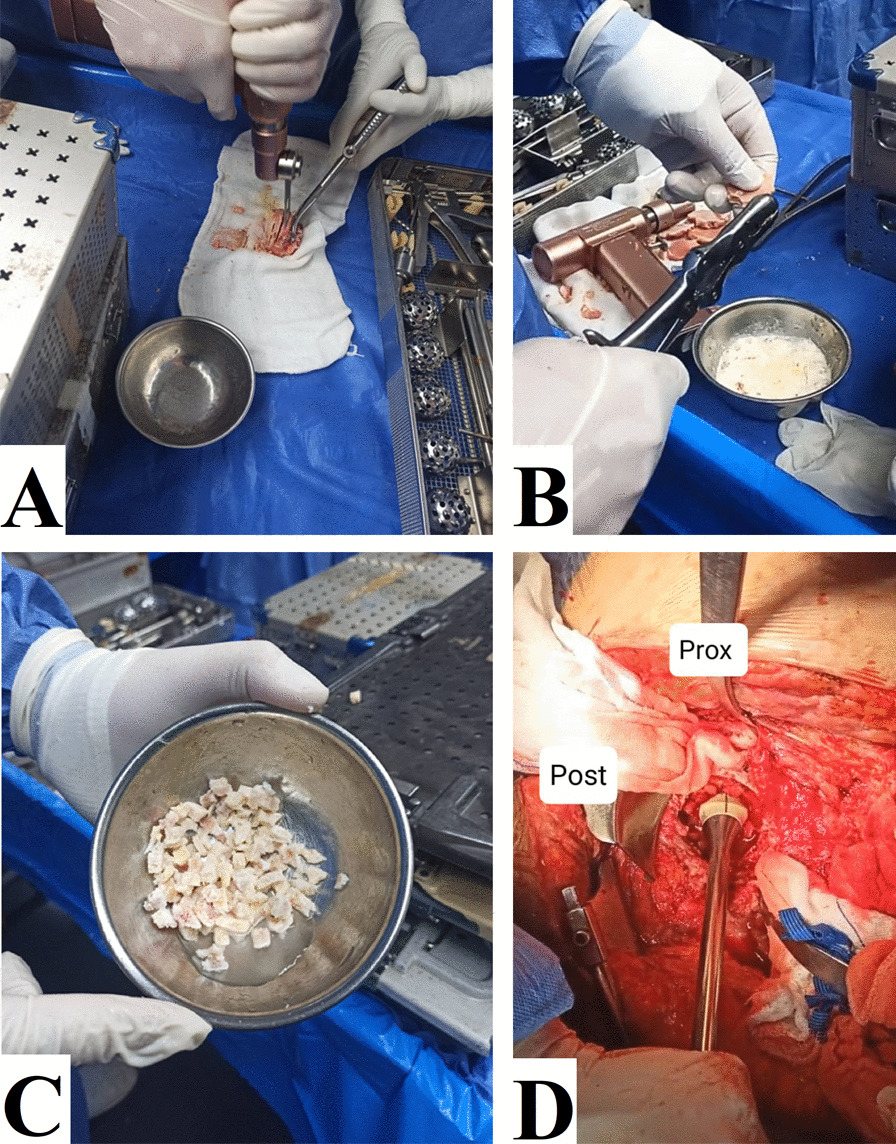
Bone graft preparation and impaction into the defect. **A** The femoral head was cut by electric saw into multiple slices. **B**, **C** The slices were cut by a bone nibbler into small morselized pieces of 0.5–1 cm in diameter. **D** The cup impactor tool was used to impact the graft into defect before insertion of the cup with or without reconstruction construct

The autogenous head of the femur was used as the IBG in primary cases. In revision cases, a cryopreserved allogeneic femoral head was used. First, the femoral head was soaked in 5% povidone–iodine solution for 30 min and cut into morselized pieces of 0.5–1-cm diameter. In allogenic grafts, the morselized bones were soaked in a 10% hydrogen peroxide solution for 15 min. The morselized bones were then washed with saline and immersed in 5-mg/ml vancomycin solution for 10 min. The morselized bones were then mixed with 2-g vancomycin. After bone bed preparation, the morselized bones were placed into the defect and impacted using the cup impactor tool, Fig. 2.

**Fig. 3:**
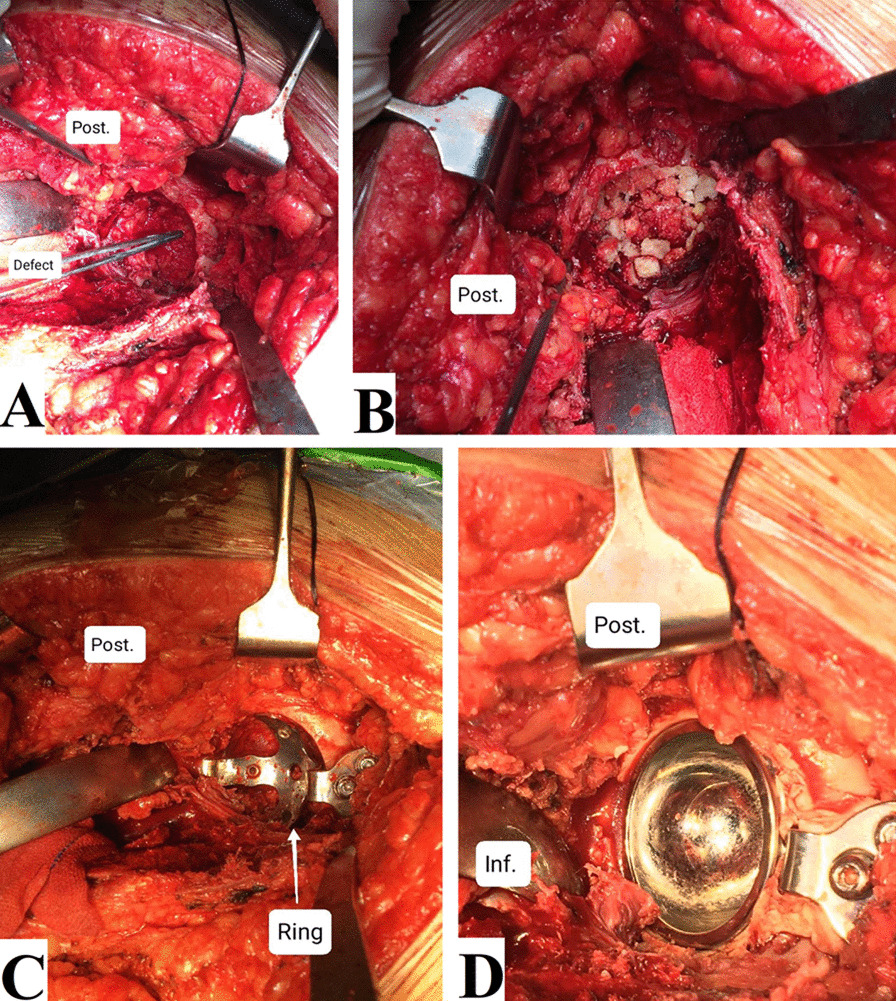
Intraoperative photographs of a revision THA case. **A** Assessment of the acetabular defect. **B** Impaction of the bone graft. **C** Placing the ring. **D** Cemented dual-mobility cup insertion

**Fig. 4:**
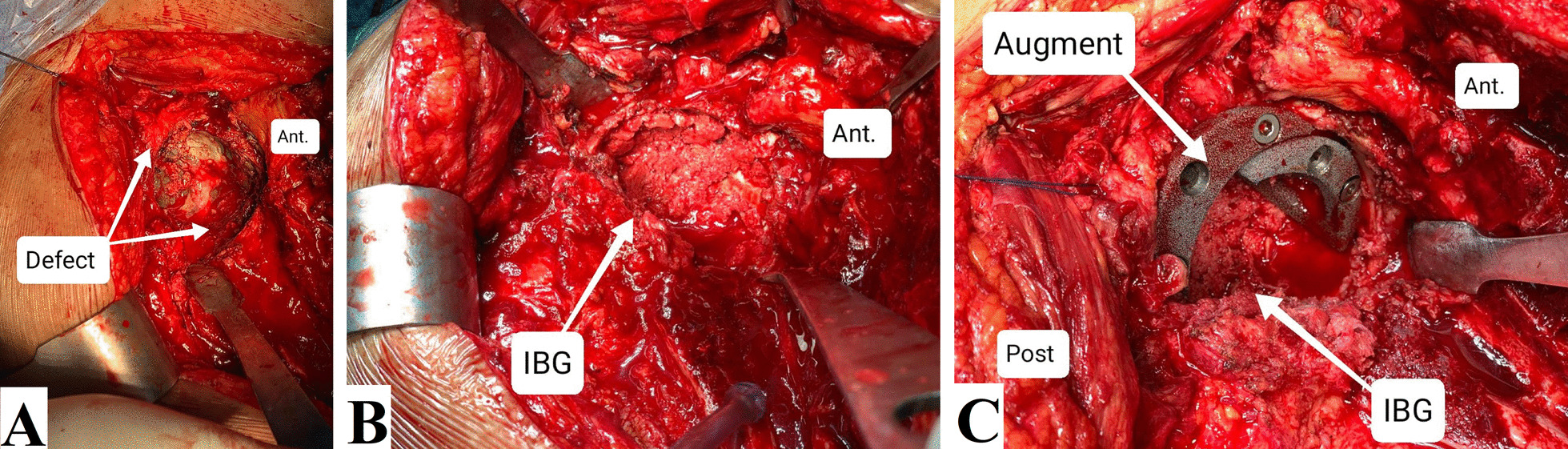
Intraoperative photographs of another revision case. **A** Assessment of the acetabular defect. **B** Impaction of the bone graft. **C** Insertion of the augment, leaving space for a primary cup

This was followed by the insertion of the acetabular cup with or without acetabular reconstruction construct, Figs. 3 and 4. In uncemented acetabular components, cup stability was achieved by press-fit fixation, and we preferred to add supplemental screws to increase the stability of the acetabular cup.

Extended trochanteric osteotomy (ETO) was used in revision cases for stem extraction and better debridement preparation of the proximal femur before insertion of the femoral stem.

A suction drain was applied, the capsule and muscles were reattached, and the wound was closed.

### Postoperative care and follow-up

On the 1st postoperative day, AP pelvis X-ray was obtained to evaluate the cup position, correction of the center of rotation, offset restoration, filling the cavitary defects with bone graft, and improvement in leg-length discrepancy. AP X-ray of the femur up to the distal extent of the stem was obtained to assess the stem position within the medullary canal and the reduction of the trochanteric osteotomy.

Postoperative antibiotic regimen was given as 1-g vancomycin intravenous infusion every 12 h for 48 h. Enoxaparin 40 IU once daily started 12 h after the surgery and maintained for 1 month.

The time to start weight bearing depended on the state of the bone stock and the quality of the component fixation. In primary cases, partial weight bearing with a walker started the 2nd day after surgery for 2 weeks. Then, two elbow crutches were used for another 4 weeks, and full weight bearing was permitted at 6 weeks postoperatively. In revision THA, where trochanteric osteotomy was done, full weight bearing was postponed to 12 weeks postoperatively.

Patients were followed up clinically and radiologically in the outpatient clinic at 6 weeks, 3 months, 1 year, and then every year postoperatively.

Clinical results were assessed utilizing the Harris hip score (HHS) [18]. Clinical outcomes were classified as excellent (HHS = 90–100), good (HHS = 80–89), fair (HHS = 70–79), and poor (HHS < 70).

Patients were categorized based on the improvement in HHS into three groups of 1–20, 21–50, and > 50 [19].

Additionally, patient satisfaction was evaluated using a short 4-question satisfaction survey [20].

**Fig. 5:**
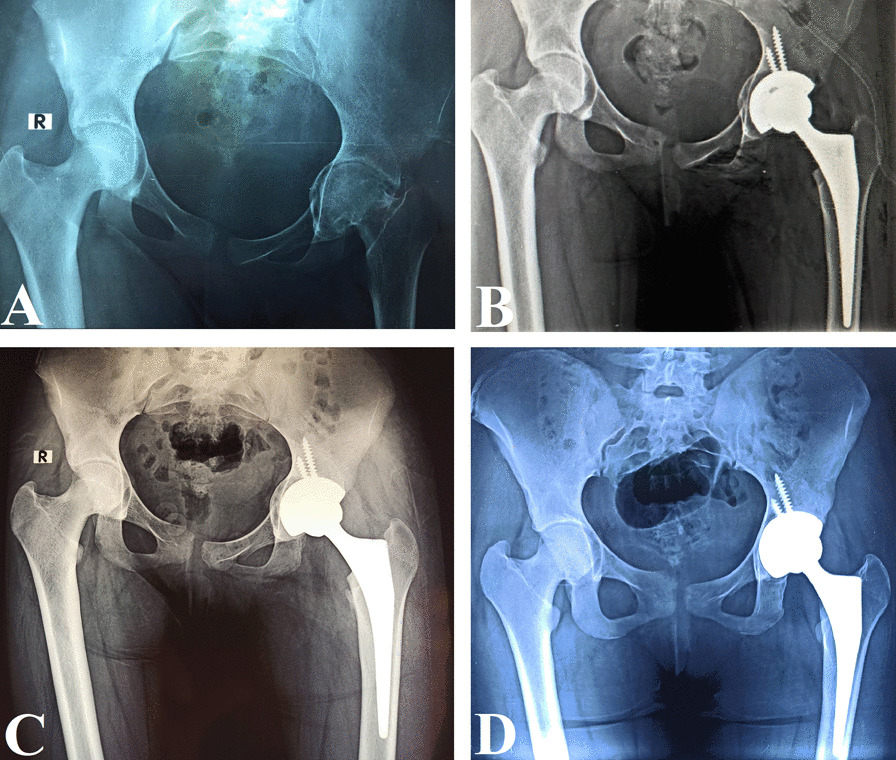
A 16-year-old female with protrusio acetabuli of the left hip treated with impaction bone grafting and primary ceramic on ceramic THA. **A** Preoperative pelvis AP X-ray. **B** Postoperative pelvis AP X-ray. **C** Six-month follow-up pelvis AP X-ray showing partial graft incorporation. **D** One-year follow-up pelvis AP X-ray showing full graft incorporation and remodeling

**Fig. 6:**
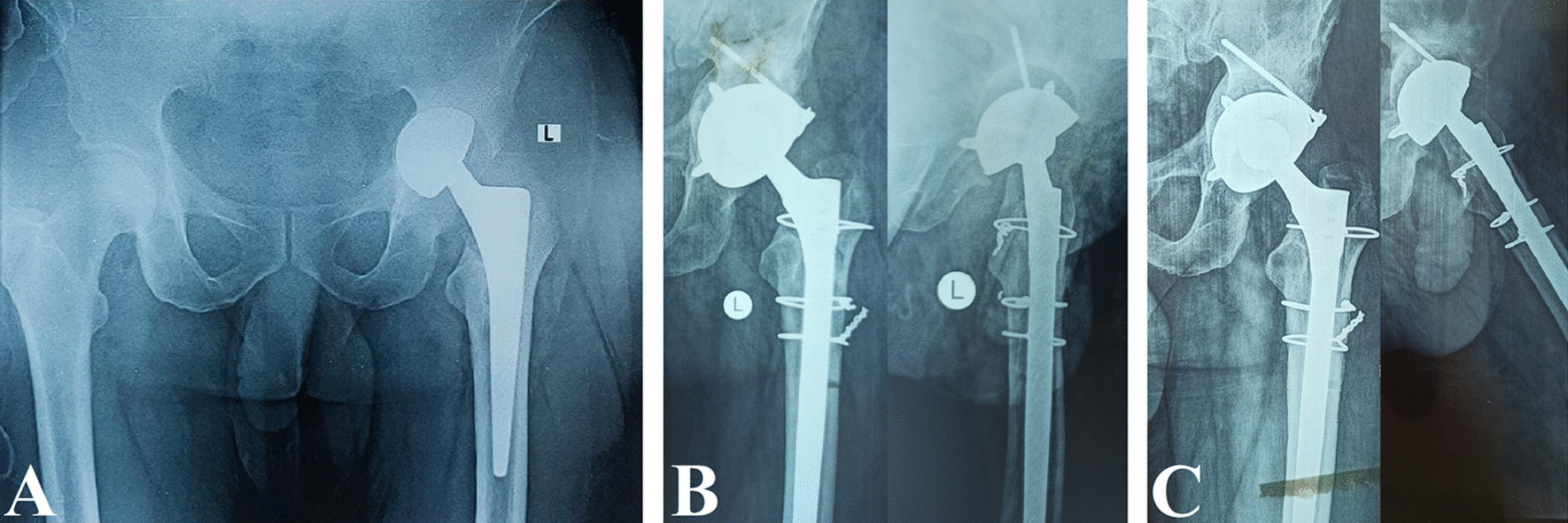
A 50-year-old male with a 5-year history of bipolar hemiarthroplasty for femoral neck fracture. The patient had aseptic loosening and cavitary acetabular defect and was treated with revision THA using long cementless stem, impaction bone grafting, and dual-mobility acetabular cup. **A** Preoperative pelvis AP X-ray. **B** Postoperative hip AP and lateral X-rays. **C** One-year follow-up hip AP and lateral X-rays showing complete graft incorporation into the host bone

**Fig. 7:**
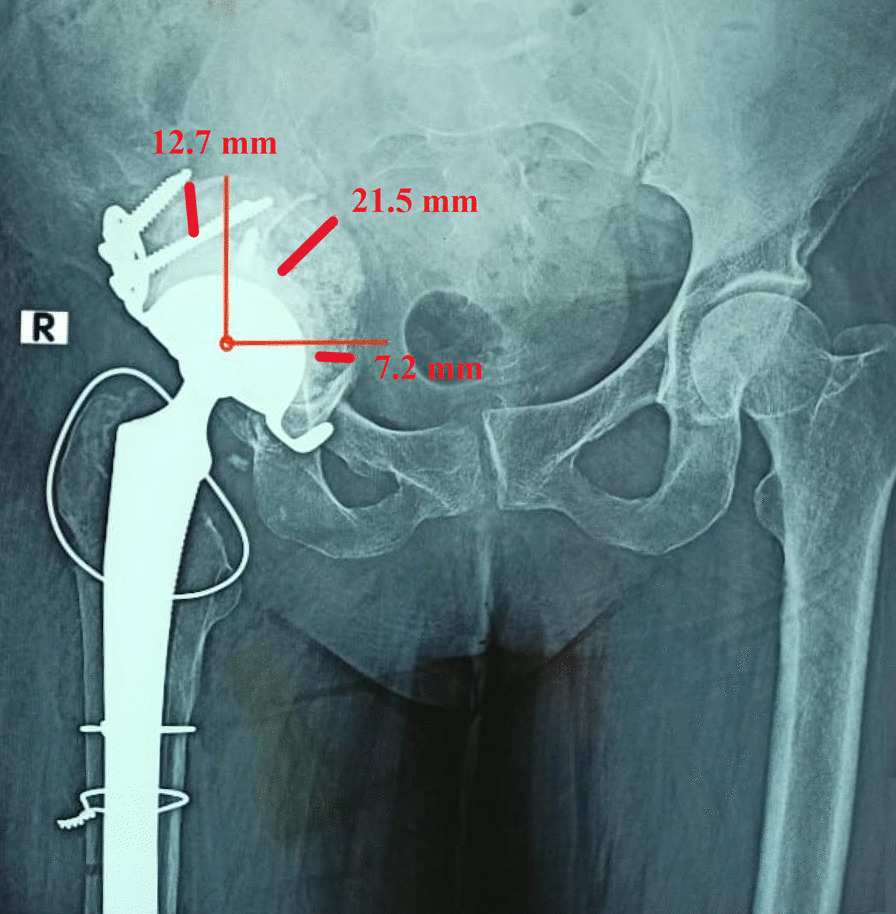
Measurement of the graft layer thickness in the widest graft area in each zone of the DeLee and Charnley acetabular zones

Bone graft incorporation and consolidation were evaluated on follow-up X-rays by three evaluators, Figs. 5 and 6. Graft incorporation was indicated by the continuation of trabecular lines from the graft into the host bone without resorption or fracture. Graft incorporation was classified at the last follow-up X-ray into complete incorporation, partial or early incorporation, no incorporation, or indistinct [21]. Additionally, graft consolidation was determined when the interface between the graft and normal bone was not identifiable with similar bone density. The graft layer thickness in the three DeLee and Charnley acetabular zones was measured at the widest graft layer in each zone, in the immediate postoperative X-rays with correction for magnification, Fig. 7. Then, the overall mean graft layer thickness in all zones was calculated.

### Statistical analysis

Data were analyzed using IBM SPSS software package version 26.0. (Armonk, NY: IBM Corp). Categorical variables were compared using the Chi-square test. Continuous variables were compared using the Student's *t*-test, Mann–Whitney test, or Wilcoxon signed-ranks test when appropriate. *P* value < 0.05 was considered significant.

## Results

### Demographics and baseline characteristics

The current study included 50 patients, 23 (46%) males and 27 (54%) females, with a mean age of 46.7 ± 15.3 (range 16–76) years. The mean body mass index (BMI) was 28.7 ± 3.7 (range 22–35) kg/m^2^. Twenty-three (46%) operations were done for the right side and 27 (54%) were for the left side.

The acetabular reconstruction was performed in conjunction with primary THA in 14 (28%) patients, while 36 (72%) patients had revision THA.

**Fig. 8:**
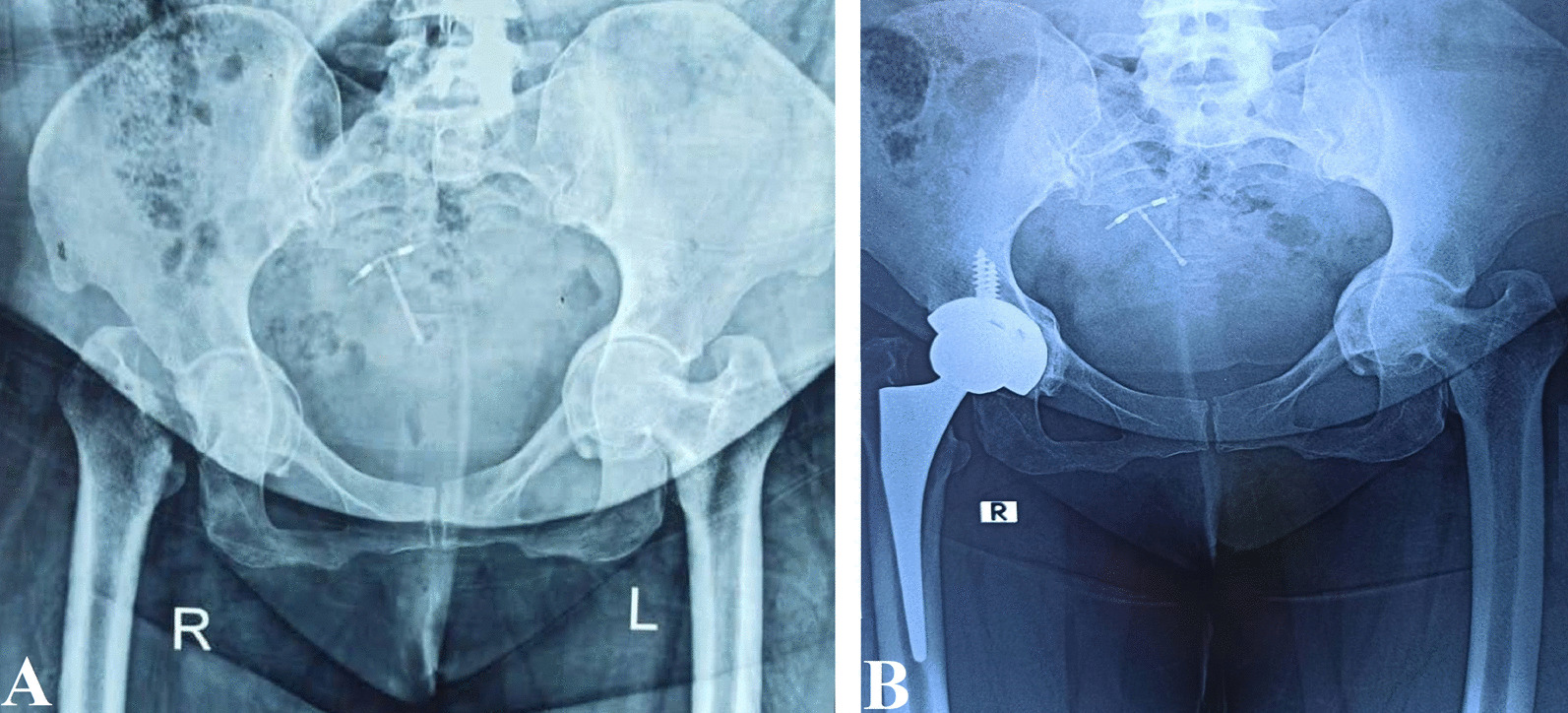
A 43-year-old female with femoral neck fracture associated with protrusio acetabuli treated with impaction bone grafting and primary cementless metal-on-polyethylene THA. **A** Preoperative pelvis AP X-ray. **B** One-year follow-up pelvis AP X-ray

The indications for primary THA were protrusio acetabuli (*n* = 8), femoral neck fractures associated with protrusio acetabuli (*n* = 4), Fig. 8, and femoral head fracture-dislocation (*n* = 2). The indications for revision THA were aseptic loosening of THR (*n* = 24), aseptic loosening of bipolar hemiarthroplasty (*n* = 6), septic loosening of THR (*n* = 4), and recurrent dislocation after THR (*n* = 2). The four patients with septic loosening were treated by two-stage revision arthroplasty.

Regarding the preoperative LLD, 26 (52%) patients had shortening of the operated limb of 11–40 mm.

The mean preoperative HHS was 28.8 ± 24.1 (range 0–65).

According to the Paprosky classification, 32 (64%) patients had type IIC acetabular defects. According to the AAOS classification, the most common type of acetabular defect was type IIB (*n* = 32, 64%), Table 1.

**Table 1:** Demographics and baseline characteristics of the included patients

Characteristics	Value (*n* = 50)
Age, years (mean ± SD)	46.7 ± 15.3
Gender (*n*, %)	
Male	23 (46%)
Female	27 (54%)
BMI, kg/m^2^ (mean ± SD)	28.7 ± 3.7
BMI categories (*n*, %)	
Underweight (< 18.5)	0 (0%)
Normal (18.5– < 25)	7 (14%)
Overweight (25– < 30)	22 (44%)
Obese (≥ 30)	21 (42%)
Affected side (*n*, %)	
Right	23 (46%)
Left	27 (54%)
Indications of surgery (*n*, %)	
Primary THR (*n* = 14)	
Protrusio acetabuli	8 (16%)
Femoral neck fractures	4 (8%)
Femoral head fracture-dislocation	2 (4%)
Secondary THR (*n* = 36)	
Aseptic loosening of THR	24 (48%)
Aseptic loosening of bipolar hemiarthroplasty	6 (12%)
Septic loosening of THR	4 (8%)
Recurrent dislocation after THR	2 (4%)
Preoperative LLD (*n*, %)	
0 mm	6 (12%)
1–10 mm	18 (36%)
11–20 mm	12 (24%)
21–30 mm	8 (16%)
31–40 mm	6 (12%)
Preoperative HHS (mean ± SD)	28.8 ± 24
Paprosky classification (*n*, %)	
Type IIA	4 (8%)
Type IIC	32 (64%)
Type IIIA	6 (12%)
Type IIIB	8 (16%)
AAOS classification (*n*, %)	
Type IIA	4 (8%)
Type IIB	32 (64%)
Type III	6 (12%)
Type IV	8 (16%)

*BMI* body mass index, *THR* total hip replacement, *LLD* leg-length discrepancy, *HHS* Harris hip score, and *AAOS* American Academy of Orthopaedic Surgeons

### Operative data

ETO was performed in 20 (40%) patients. The femoral stem was revised in 38 (76%) patients. Acetabular reconstruction using augment or ring was done in 14 (28%) patients. The most commonly used cups were cementless augmented dual-mobility cups (*n* = 18, 36%) and cemented dual-mobility cups (*n* = 16, 32%), Table 2.

**Table 2:** Operative details of the included patients

Characteristics	Value (*n* = 50)
ETO (*n*, %)	
Yes	20 (40%)
No	30 (60%)
Femoral stem (*n*, %)	
Not revised	12 (24%)
Cementless primary stem	16 (32%)
Cementless long stem non-locked	10 (20%)
Cementless long stem locked	12 (24%)
Acetabular reconstruction (*n*, %)	
No	36 (72%)
Augment	2 (4%)
Kerboul cross-ring	11 (22%)
Kerboul cross-ring and plate	1 (2%)
Types of acetabular cups (*n*, %)	
Cementless CoP	4 (8%)
Cementless MoP	4 (8%)
Cementless dual-mobility cup	8 (16%)
Cementless augmented dual-mobility cup	18 (36%)
Cemented dual-mobility cup	16 (32%)

*ETO* extended trochanteric osteotomy, *CoP* ceramic on polyethylene, and *MoP* metal on polyethylene

### Functional and radiological outcomes

The mean follow-up period was 23 (range 12–34) months.

The mean HHS increased significantly to 76.6 ± 6.1 (range 60–87) at the last follow-up visit, *P* < 0.001. The results were good in 12 (24%) patients, fair in 30 (60%) patients, and poor in 8 (16%) patients. Regarding the HSS improvement, 30 (60%) patients had improvement of 21–50, and 20 (40%) patients had improvement of > 50.

The mean BMI was higher in patients with improvement of > 50 points in the HHS (30.7 ± 2.7) than in patients with improvement of 21–50 points, 27.3 ± 3.7, *P* < 0.001. In patients with > 50 points improvement in the HHS, 80% did not have ETO, *P* = 0.018, Table 3.

**Table 3:** Relation between Harris hip score (HHS) improvement and different variables

Variables	HHS improvement		*P* value
	21–50 (*n* = 30)	> 50 (*n* = 20)	
Gender (*n*, %)			
Male	14 (46.7%)	9 (45%)	0.908
Female	16 (53.3%)	11 (55%)	
BMI, kg/m^2^ (mean ± SD)	27.3 ± 3.7	30.7 ± 2.7	** < 0.001**
BMI categories (*n*, %)			
Underweight (< 18.5)	0 (0%)	0 (0%)	**0.018**
Normal (18.5– < 25)	7 (23.3%)	0 (0%)	
Overweight (25– < 30)	14 (46.7%)	8 (40%)	
Obese (≥ 30)	9 (30%)	12 (60.0%)	
Type of arthroplasty (*n*, %)			
Primary	8 (26.7%)	6 (30%)	0.797
Revision	22 (73.3%)	14 (70%)	
Paprosky classification (*n*, %)			
Type II	22 (73.3%)	14 (70%)	0.797
Type III	8 (26.7%)	6 (30%)	
ETO (*n*, %)			
Yes	16 (53.3%)	4 (20%)	**0.018**
No	14 (46.7%)	16 (80%)	

*BMI* body mass index and *ETO* extended trochanteric osteotomy

Bold indicates *P* value < 0.05 is statistically significant

At the last follow-up, patient satisfaction was graded as high (very satisfied) in 44 (88%) patients, moderate (somewhat satisfied) in 4 (8%) patients, and low (somewhat dissatisfied) in 2 (4%) patients.

Postoperatively, the LLD was within 0–5 mm shortening in 40 (80%) patients, 6–10 mm shortening in 4 (8%) patients, 11–20 mm shortening in 4 (8%) patients, and 1–5 mm lengthening in 2 (4%) patients.

Regarding the radiological graft incorporation to host bone, 35 (70%) patients had complete incorporation, and 15 (30%) patients had partial incorporation. Partial incorporation was noticed in Delee and Charnley zone 3 (*n* = 12) and zone 2 (*n* = 3). Graft consolidation was confirmed in 28 (56%) patients.

Additionally, the mean thickness of the graft layer in DeLee and Charnley zones was 11.3 ± 4.1 (range 5–22) mm in zone 1, 12.5 ± 4.3 (range 6–23) mm in zone 2, and 10.8 ± 3.3 (range 6–18) mm in zone 3. The mean overall graft thickness in all zones was 11.7 ± 3.7 (range 5–21) mm. All patients had re-trabeculation and remodeling of the impacted graft.

All patients with partial graft incorporation had revision arthroplasty with impaction allograft, *P* = 0.004. Complete graft incorporation was more commonly achieved with cementless cup compared to the cemented cup, *P* < 0.001. Based on Paprosky and AAOS classifications, the smaller the size of the acetabular defect, the more likely to achieve complete graft incorporation, *P* < 0.001. The average thickness of the graft layer in patients with partial incorporation was higher than in patients with complete incorporation, 15.5 ± 3.8 and 10 ± 2.2 mm, respectively, *P* < 0.001, Table 4.

**Table 4:** Relation between radiological graft incorporation into host bone and different variables

Variables	Radiological graft incorporation		*P* value
	Partial or early incorporation (*n* = 15)	Complete incorporation (*n* = 35)	
Age, years (mean ± SD)	48.3 ± 18.4	45.9 ± 14.1	0.618
Gender (*n*, %)			
Male	5 (33.3%)	18 (51.4%)	0.239
Female	10 (66.7%)	17 (48.6%)	
History of infection (*n*, %)			
No	15 (100)	31 (88.6%)	0.302
Yes	0 (0)	4 (11.4%)	
Type of arthroplasty (*n*, %)			
Primary	0 (0%)	14 (40%)	**0.004**
Revision	15 (100%)	21 (60%)	
Type of graft (*n*, %)			
Autograft	0 (0%)	14 (40%)	**0.004**
Allograft	15 (100%)	21 (60%)	
Cemented or cementless cup (*n*, %)			
Cemented	11 (73.3%)	5 (14.3%)	** < 0.001**
Cementless	4 (26.7%)	30 (85.7%)	
Paprosky classification (*n*, %)			
Type II	2 (13.3%)	34 (97.1%)	** < 0.001**
Type III	13 (86.7%)	1 (2.9%)	
AAOS classification (*n*, %)			
Type IIA	0 (0.0%)	4 (11.4%)	** < 0.001**
Type IIB	2 (13.3%)	30 (85.7%)	
Type III	5 (33.3%)	1 (2.9%)	
Type IV	8 (53.3%)	0 (0.0%)	
Average thickness of graft layer, mm (mean ± SD)	15.5 ± 3.8	10 ± 2.2	** < 0.001**

*AAOS* American Academy of Orthopaedic Surgeons

Bold indicates *P* value < 0.05 is statistically significant

### Complications

Intraoperative complications included iatrogenic acetabular defect (*n* = 1) treated immediately by conversion of the cup to augmented dual-mobility cup with flanges fixed by screws, greater trochanteric fracture (*n* = 1), and acetabular perforation with reaming were no affection of cup stability (*n* = 1). Postoperative complications included sciatic nerve traction palsy (*n* = 2), which was resolved spontaneously in 4 weeks.

## Discussion

Acetabular bone defects are frequently encountered in THA, and the reconstruction can be challenging for surgeons, especially in large defects [22].

In this study, we evaluated the clinical and radiological outcomes of IBG for the reconstruction of acetabular defects in primary and revision THA. The mean HHS improved significantly with a mean follow-up of 23 months. Overall, 40% of patients had an improvement of the HHS of more than 50 points. Complete graft incorporation was achieved in 70% of patients, and all patients had re-trabeculation and remodeling. Factors that were associated with complete graft incorporation included primary THA, use of autograft, cementless cup, decreased defect size based on Paprosky and AAOS classifications, and decreased thickness of graft layer. The acetabular cup constructs remained stable with no signs of subsidence or migration through the follow-up period, and no patients required revision surgery.

In primary and revision THA, IBG has been reported to have excellent clinical and radiographic outcomes with a high implant survival rate [13, 23]. Özdemir et al. [24] reported favorable long-term outcomes of cemented primary THA combined with IBG in patients younger than 25 years with acetabular bone deficiencies. Welten et al. [2] reported a mean follow-up HHS of 88, after using impaction autograft and cemented primary THA. Significant improvement in HHS was reported by van Egmond et al. [25], with a 10-year survival rate of 88% in patients with large acetabular defects reconstructed with IBG and a cemented cup.

In our study, patients with > 50 points improvement in the HHS had significantly higher BMI, and 80% of them did not have ETO. However, to confirm these findings, a large sample size and multivariable analysis should be performed.

Buttaro et al. [12] reported 90.8% cup survival after using IBG, metal mesh, and cemented cup in patients with cavitary uncontained acetabular defects in revision THA, with a mean follow-up of 36 months. Schreurs et al. [26] reported 96% and 84% cup survival at 10 and 15 years of follow-up, respectively, in patients undergoing acetabular revision with IBG and cemented cups. Schreurs et al. [10] in another study described favorable outcomes of subsequent acetabular re-revisions with IBG and cemented cups following a prior acetabular revision with the same procedure.

Our study had six traumatic cases with femoral neck fractures or femoral head fracture-dislocation. These cases were operated on within the 1st week of trauma. The used IBG was autogenous femoral heads. At the last follow-up, we did not notice any necrosis or graft resorption.

In our study, IBG incorporation with host bone was significantly less optimal in large defects. Buttaro et al. [12] reported favorable results in acetabular deficiencies of medium severity and less successful results in more extensive combined deficiencies. van Haaren et al. [27] evaluated the outcomes of using IBG for large deficiencies and reported a high rate of failure, but the used graft in all patients was allograft not autograft.

Graft incorporation is a dynamic biological process that includes a series of events of inflammation, revascularization, the substitution of the graft with new bone, and remodeling [28]. In our study, 70% of patients had complete graft incorporation with a mean follow-up of 23 months. All autografts incorporated completely into host bone, with a significant relationship between the type of graft and the graft incorporation. In patients with dysplastic hip who underwent cementless THA, Mozafari et al. [22] reported 96.5% rate of complete graft incorporation in patients with dysplastic hips with over 30% acetabular bone defect who had impaction autograft and cementless THA, with a mean follow-up of 93.3 months.

In our study, there was a significant relationship between partial graft incorporation to host bone and increased thickness of the graft layer. van Haaren et al. [27] reported that the graft layer thickness was higher in patients who required revision for aseptic loosening.

In the current study, full graft incorporation was noticed in patients who had second stage revision for septic loosening, was no significant relationship between history of infection and incorporation to host bone. Hsieh et al. [29] reported full allograft incorporation into host bone in all patients in their study of patients with two-stage revision THA with extensive bone loss.

No graft resorption was noticed in the study. However, this remains a formidable challenge as graft resorption may occur over time and cause implant loosening. van Haaren et al. [27] reported a high failure rate with IBG in large acetabular deficiencies. Recently, some authors recommended the use of combined IBG with augments. De la Torre-Escuredo et al. [30] reported that using combined IBG with trabecular metal augments yielded satisfactory outcomes in young adults with extensive acetabular defects. There was no significant difference in abduction angle or cup migration at the last follow-up compared to the immediate postoperative radiograph [30]. Similarly, Gill et al. [31] reported no failure after using IBG combined with trabecular metal augments in 15 acetabular defects of Paprosky types 2B and 3A.

In our study, the morselized graft size was 0.5–1-cm size pieces. Welten et al. [2] used similar graft size and reported good long-term results, with a mean follow-up of 12.3 years. Holton et al. [32] compared three groups of bone chip size of 2–4 mm^3^, 10 mm^3^, and 20 mm^3^ and reported that the 10-mm^3^ size was optimal in providing initial mechanical stability. Mirza and Sadiq [33] recommended preparing the bone chips manually with a rongeur with an optimal size of 8–10 mm.

Replenishing the bone stock with IBG remains an optimal technique to address acetabular bone loss, with reliable, excellent clinical, and radiological results. Placing the acetabular component in its original position is crucial and reduces the risk of dislocation and loosening [34, 35].

This study has some limitations, including the absence of a control group, and the wide diversity of cases such as using autograft or allograft and primary or revision THA. We were unable to separate primary and revision THA cases due to the relatively small number of patients. Additionally, we did not quantify the amount of bone graft intra- or postoperatively. Also, multivariable analysis was not performed when assessing the factors affecting the rate of HHS improvement. Finally, the follow-up period was relatively short and inadequate to exclude late complications such as component loosening. However, it was long enough to assess IBG incorporation and its influencing factors.

## Conclusion

IBG for acetabular reconstruction in THA can achieve satisfactory results with high rate of radiological graft incorporation and low complication rate. Factors associated with favorable graft incorporation include primary THA, autografts, cementless cups, reduced defect size, and decreased thickness of the graft layer.

## Data Availability

The dataset analyzed in this study is available from the corresponding author on request.
